# Mental Well-Being and the Quality of Life Among Retired Public and Private Sector Employees: A Comparative Study From Kerala, India

**DOI:** 10.7759/cureus.71663

**Published:** 2024-10-16

**Authors:** Muralidharan K Pranav, Paul T Francis, Jeby Jose Olickal, Brilly M Rose, P. Sankara Sarma, Kavumpurathu R Thankappan

**Affiliations:** 1 Department of Public Health, Amrita Institute of Medical Sciences, Kochi, IND; 2 Department of Community Medicine, Amrita Institute of Medical Sciences, Kochi, IND

**Keywords:** comparative study, mental well-being, older adults, public health problems, retirement

## Abstract

Background

Retirement from an active working environment is one of the important risk factors for mental health problems. The literature on the mental well-being and quality of life among retired public and private sector employees in Kerala is limited. We conducted this study to compare the mental well-being, quality of life and factors associated with them among retired public and private sector employees in Kollam district, Kerala.

Methods

This community-based cross-sectional study was conducted among 320 retired employees (mean age 69.5 years, Men 172 (53.8%), public sector employees 160 (50%)) selected using multistage cluster sampling. The Warwick-Edinburgh Mental Well-being Scale (WEMWBS) and WHO QoL BREF questionnaire were used for data collection. Multivariate analyses were used to find out the factors associated with mental well-being and quality of life.

Results

Among the retired private employees, 94 (58.75%) and those from the public sector three (1.88%) reported low mental well-being (Adjusted prevalence ratio (APR) =21.28, 95% CI: 6.34-71.36, p=<0.001). Participants aged > 68 years (APR=1.36, 95% CI: 1.03-1.79, p=0.026) and rural residents (APR= 1.47, 95% CI: 1.09-1.97, p=0.016) reported lower mental well-being compared to their counterparts. Retired public sector employees' quality of life was higher (Adjusted Mean Difference (AMD) =18.67, 95% CI: 15.48-21.86, p=<0.001) compared to retired private sector employees. Participants aged <= 68 years (AMD=7.01, 95% CI: 4.49-9.52, p=<0.001), male gender (AMD=4.25, 95% CI: 1.68-6.81, p=0.001), rural residents (AMD= 2.6, 95% CI: 0.08-5.13, p=0.043) and belonging to above poverty line (AMD=8.12, 95% CI: 4.08-12.16, p=<0.001) had higher QoL scores.

Conclusion

Efforts are required to improve retirement policies that support the mental health and quality of life of retired private sector employees.

## Introduction

The retirement age in India ranks among the lowest globally. While many countries have established a retirement age ranging from 66 to 67 years, in India, the retirement age for private sector employees falls between 58 and 60 years [1]. Public sector employees in India typically retire at 60 years, except in Kerala, where the retirement age is 56. Elderly households in India are particularly vulnerable to financial shocks due to a lack of a strong and universal social security system, low coverage of old-age pensions, a high percentage of employment in the unorganized sector, early retirement from formal employment, and rising health care costs [2].

Retirement marks the end of an individual's employment and signifies a major life transition [3]. It involves withdrawal from one's position, occupation, or active working life [4]. This significant adjustment can have an effect on mental health, potentially leading to both positive and negative outcomes [5]. According to the World Health Organization (WHO), 14% of adults aged 60 and above live with a mental disorder, and according to Global Health Estimate 2019, it accounts for 10.6% of total disability (DALY) among older adults. Older adults experience a drop in income or a reduced sense of purpose with retirement, which can lead to serious mental health problems [6]. Early recognition, diagnosis, and proper care of mental health problems in older adults are important to reduce their suffering and disabilities [7].

Retired public and private sector employees are subject to different structures, work cultures, and job demands. Retired public sector employees generally include individuals who receive higher work compensations, such as pensions, compared to retired private sector employees. Although studies examined stress factors and workplace health among public and private employees, there is a scarcity of comparative research on the mental well-being and quality of life among retired public and private sector employees. Therefore, in this study, we aimed to compare the mental well-being and quality of life of retired public and private sector employees in Kerala, India.

## Materials and methods

Study design and setting

This is a community-based cross-sectional study conducted in Kollam, a southern district of Kerala state, India. This study was conducted among retired employees aged 60 years and above, excluding those who were bedridden or unable to respond. The study period was six months, from November 2023 to May 2024. In Kerala, retired public sector employees benefit from a lifetime employment pension scheme. For private sector retirees, the primary social security option is the National Pension Scheme (NPS), which allows for a contributory pension plan with structured withdrawal options upon retirement, including a combination of lump sum withdrawal and annuity purchase to generate regular income. Additionally, the Employee Provident Fund (EPF) also provides pension benefits to private sector employees based on their years of service, offering crucial financial security measures for retirees. There are a few more social security schemes for the elderly population in Kerala. The ‘Vayomithram’ initiative, implemented by the Kerala Social Security Mission, provides support and medical care to senior citizens over the age of 65 years living in Corporation/Municipal areas of the state. The Vayomithram project primarily offers free medications to the elderly through help desks, palliative care, and mobile clinics [8]. Additionally, the Indira Gandhi National Old Age Pension Scheme, sanctioned by the government as a social security measure to aid the economically backward and helpless in society, currently provides beneficiaries with a monthly pension amount of Rs 1600/- (Approx. USD 20) [9].

Sample size calculation

Based on the mean ± SD of mental well-being score observed in a pilot study with 30 participants, retired public sector employees had a score of 51±7.93 while retired private employees had a score of 46.29±9.64. With a 90% confidence level, the minimum calculated sample size was 146. Considering a design effect of 2, the sample size was increased to 292. To accommodate 20 participants from 16 clusters, the final sample size used for the study was 320.

Sampling method

**Figure 1:**
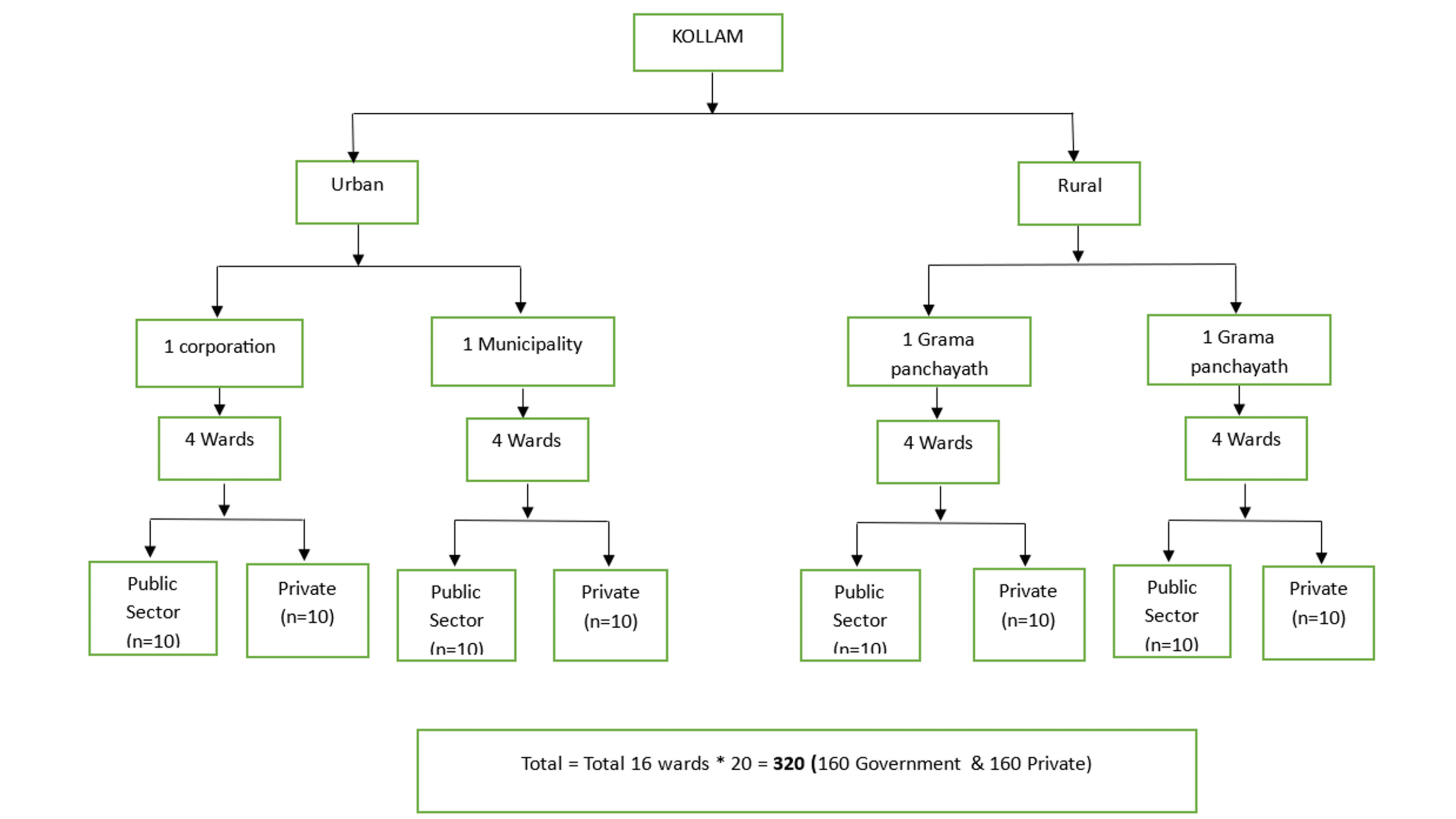
Flowchart depicting sampling technique

Multistage cluster sampling was used in this study. From the urban area of Kollam district, one corporation and one municipality (out of four) were selected using the lottery method. Similarly, out of 69 Gramapanchayaths of rural areas, two Gramapanchayaths were selected using Microsoft Excel for random sampling. From each of the selected corporations, municipalities, and Gramapanchayaths, four wards were selected randomly (totaling 16 wards). From each ward, a direction was chosen randomly, and all the right-sided houses were surveyed. The household survey continued until 10 retired public sector and 10 retired private sector employees were recruited. The detailed sampling technique is depicted in Figure 1.

Study tools and process of administration

After obtaining clearance from the institute ethics committee, the survey was conducted using a semi-structured questionnaire. Written informed consent was taken before enrolment. The questionnaire had three sections: Section I covered sociodemographic and behavioral factors, Section II focused on the mental well-being of the study participants, and Section III addressed quality of life. The mental well-being of the study participants was assessed using the Warwick-Edinburgh Mental Well-being Scale (WEMWBS), and quality of life was assessed using the World Health Organization (WHO) Quality of Life (QoL) BREF questionnaire.

The Warwick-Edinburgh Mental Well-being Scale (WEMWBS) is a 14-item scale used to measure mental wellbeing. The scale is scored by summing the responses to each item, which are answered on a 1-5 Likert scale. The minimum score is 14, and the maximum is 70. Scores are categorized into high (60-70), medium (43-59), and low (14-42).

The WHO QoL BREF questionnaire comprises 26 questions divided into four domains: physical, psychological, environmental, and social. The scores for the four domains explain the perception of quality of life in each specific domain. Domaines scores are in a positive direction, with higher scores indicating a higher quality of life. The mean scores are calculated for each domain and then multiplied by four to make it comparable with the WHO QoL-100.

Study variables

Independent variables: Age, gender, religion, marital status, area of residence, socioeconomic status; assessed using the type of ration cards and categorized as APL (Above Poverty Line) and BPL (Below Poverty Line), education, past employment, years of work, retirement income, job satisfaction during past employment, alcohol use, and tobacco use. Dependent variables: Mental well-being (low, medium, and high) and Quality of life (four domains - physical, psychological, social, and environmental)

Statistical analysis

Data were entered using EpiData version 4 (www.epidata.dk) and statistical analysis was performed using STATA version 14 (StataCorp LLC, College Station, TX). Categorical variables were expressed as frequencies and percentages, and continuous variables were presented as mean ± standard deviation or median (quartile range). The normality of the data was assessed using the Kolmogorov-Smirnov test. Mental well-being was compared using the Chi-square test and unadjusted prevalence ratios (UPR) with 95% confidence intervals (CIs) were calculated using univariate regression analysis. Variables that had a p value less than 0.2 in the univariate were included in the multivariable analysis using the log-binomial model to estimate the adjusted PR (APR) with 95% CI. QoL scores between retired public and private sector employees were compared using the Mann-Whitney U test for two groups and the Kruskal-Wallis test for three groups. Multivariable linear regression analysis was used to predict factors determining QoL and adjusted mean differences (AMD) with 95% CI were calculated. Statistical significance was considered at p < 0.05.

## Results

Of the total participants, half were aged above 68 years, 168 (46.2%) were females, 285 (89.1%) were Hindus, and 126 (39.3%) were currently married. The details of socioeconomic characteristics are depicted in Table 1.

**Table 1:** Socio-demographic characteristics of the study participants (N=320) APL-Above poverty line, BPL-Below poverty line.

Variables	Number	Percentage
Age (years)		
≤ 68	162	50.6
> 68	158	49.4
Sex		
Male	172	53.8
Female	148	46.2
Religion		
Hindu	285	89.1
Christian	25	7.8
Muslim	10	3.1
Marital status		
Married	126	39.3
Others	194	60.7
Area of residence		
Urban	160	50.0
Rural	160	50.0
Socioeconomic status		
APL	277	86.6
BPL	43	13.4
Highest educational qualification		
Below primary school	32	10.0
Primary to higher secondary	131	40.9
Degree and above	157	49.1

Table 2 shows the participants' distribution based on employment and behavioral characteristics. Of the participants, 143 (44.7%) had more than 25 years of experience, and 199 (62.2%) were satisfied with their past employment. About 21 (6.6%) were current alcohol users, and 12(3.8%) were current tobacco users.

**Table 2:** Pattern of employment and behavioural characteristics of the study participants (N=320)

Variables	Frequency	Percentage
Retired from		
Private sector	160	50.0
Public sector	160	50.0
Years of work		
≤25	177	55.3
>25	143	44.7
Retirement income ( employment pension)		
Yes	160	50.0
No	160	50.0
Past job satisfaction		
Good	199	62.2
Bad	121	37.8
Alcohol use		
Yes	21	6.6
No	299	93.4
Tobacco use		
Yes	12	3.8
No	308	96.3

The prevalence of low mental well-being was 30.3% (n=97). The median (IQR) QoL domain scores were: social health, 50 (41.67-75.0); psychological health, 62.5 (54.17-79.17); physical health, 64.3 (46.43-75.0); and environmental health, 65.63 (50-75).

Table 3 shows factors associated with low mental well-being. The table indicates that 94 (58.75%) of retired private sector employees reported low mental well-being compared to only 3 (1.88%) of public sector employees, with a 21.28 times higher likelihood of low mental well-being (95% CI: 6.34-71.36). Participants over 68 years of age had a lower level of mental well-being 61 (38.61%) than participants =<68 years of age 36 (22.22%), with a 1.36 higher likelihood of lower mental well-being (95% CI: 1.03-1.79). Participants living in rural areas had a higher likelihood of lower mental well-being (APR=1.47, 95% CI 1.09-1.97) compared to those in urban areas.

**Table 3:** Factors associated with low mental well-being Result of bivariate and multivariable regression analysis (N=320). APL-Above poverty line, BPL-Below poverty line, * Variables that had a p-value <0.2 in the unadjusted analysis were used for the adjusted analysis, NA-Not applicable

Variables	Low mental well-being		Unadjusted Prevalence Ratio (95% Confidence Interval)	Adjusted Prevalence Ratio (95% Confidence Interval)	p-value*
	Yes	No			
	n(%)	n(%)			
Retired from					
Private sector	94 (58.75)	66 (41.25)	33.33(10.13-96.84)	21.28 (6.34-71.36)	<0.001
Public sector	3 (1.88)	157 (98.12)	Reference	Reference	NA
Age (years)					
> 68	61 (38.61)	97 (61.39)	1.73(1.22-2.46)	1.36 (1.03-1.79)	0.026
≤ 68	36 (22.22)	126 (77.78)	Reference	Reference	NA
Sex					
Female	62 (41.89)	86 (58.11)	2.05(1.44-2.92)	1.20 (0.91-1.59)	0.181
Male	35 (20.35)	137 (79.65)	Reference	Reference	NA
Religion					
Hindu	89 (31.23)	196 (68.77)	2.60 (0.88-7.62)	1.94 (0.64-5.86)	0.236
Muslim	5 (50.0)	5 (50.0)	4.16(1.21-14.24)	2.40 (0.71-8.10)	0.158
Christian	3 (12.0)	22 (88.0)	Reference	Reference	NA
Marital status					
Married	37 (29.37)	59 (70.63)	Reference	NA	NA
Others	60 (30.93)	134 (69.07)	0.80(0.57-1.11)	NA	NA
Area of Residence					
Rural	59 (36.88)	101 (63.12)	1.55(1.10-2.18)	1.47 (1.09-1.97)	0.016
Urban	38 (23.75)	122 (76.25)	Reference	Reference	NA
Socioeconomic status					
BPL	26 (60.47)	17 (39.53)	2.35(1.72-3.22)	0.97 (0.72-1.29)	0.842
APL	71 (25.63)	206 (74.37)	Reference	Reference	NA
Highest educational qualification					
Below primary school	20 (62.50)	12 (37.50)	4.51(2.55-7.99)	1.61 (0.96-2.67)	0.066
Primary to higher secondary	65 (41.40)	92 (58.60)	6.82(3.73-12.46)	1.57 (0.91-2.71)	0.101
Degree and above	12 (9.16)	119 (90.84)	Reference	Reference	NA
Years of work					
≤25	76 (42.94)	101 (57.06)	2.92(1.90-4.49)	1.24 (0.90-1.72)	0.177
>25	21 (14.69)	122 (85.31)	Reference	Reference	NA
Alcohol use					
Yes	5 (23.81)	16 (76.19)	Reference	NA	NA
No	92 (30.77)	207 (69.23)	1.29(0.59-2.82)	NA	NA
Tobacco use					
Yes	3 (25.00)	9 (75.00)	Reference	NA	NA
No	94 (30.52)	214 (69.48)	1.22(0.45-3.29)	NA	NA

Table 4 shows factors associated with QoL. The AMD in QoL was higher among retired public sector employees (AMD=18.67, 95% CI: 15.48-21.86) compared to retired private sector employees. Participants aged 68 years and below (AMD=7.01, 95% CI: 4.49-9.52), male gender (AMD=4.25, 95% CI: 1.68-6.81), those belonging to rural areas (AMD= 2.6, 95% CI: 0.08-5.13) and those belonging to APL (AMD=8.12, 95% CI: 4.08-12.16) also had higher QoL scores compared to their counterparts.

**Table 4:** Factors associated with the quality of life Result of bivariate and multivariate analysis (N=320). APL-Above poverty line, BPL-Below poverty line, * Variables that had a p-value <0.2 in the unadjusted analysis were used for the adjusted analysis, NA-Not applicable

Independent Variables	Total score	p-value	Adjusted mean difference (95% Confidence Interval)	p-value*
	Median (Q1-Q3)			
Retired from				
Public sector	68.04 (23.96-100)	<0.001	18.67 ( 15.48 - 21.86 )	<0.001
Private sector	56.40 (20.83-92.86)	NA	Reference	NA
Age (years)				
≤ 68	68.04 (23.96-100)	<0.001	7.01 ( 4.49 – 9.52 )	<0.001
> 68	56.40 (20.83-92.86)	NA	Reference	NA
Gender				
Male	67.89 (23.96-100)	<0.001	4.25 (1.68 - 6.81 )	0.001
Female	54.76 (20.83-95.63)	NA	Reference	NA
Religion				
Hindu	63.62 (20.83-95.83)	0.263	NA	NA
Christian	62.13 (32.55-100)	NA	NA	NA
Muslim	52.01 (22.77-71.88)	NA	NA	NA
Marital status				
Married	64.95 (20.83-100)	0.379	NA	NA
Others	61.83 (22.76-94.41)	NA	NA	NA
Area of Residence				
Urban	64.08 (22.77-95.83)	0.056	2.60 ( 0.08 - 5.13 )	0.043
Rural	61.59 (20.83-100)	NA	Reference	NA
Socioeconomic status				
APL	65.29 (22.77-100)	<0.001	8.12 ( 4.08 - 12.16 )	<0.001
BPL	43.08 (20.83-75.11)	NA	Reference	NA
Education				
Primary schooling to higher secondary	56.44 (22.77-88.69)	<0.001	0.61 ( -3.91 – 5.15 )	0.789
College education and above	71.65 (28.57-100)	<0.001	4.72 ( -0.27 - 9.73 )	0.064
Below primary schooling	47.23 (20.83-75.11)	NA	Reference	NA
Years of work				
>25	69.79 (20.83-100)	<0.001	1.02 (-1.81 – 3.86 )	<0.479
≤25	55.25 (22.77-90.55)	NA	Reference	NA
Alcohol use				
Yes	66.59 (29.61-90.40)	0.263	NA	NA
No	62.28 (20.83-100)	NA	NA	NA
Tobacco use				
Yes	71.35(29.61-87.28)	0.47	NA	NA
No	63.08(20.83-100)	NA	NA	NA

## Discussion

In the current study, we found that retired public sector employees had higher levels of mental well-being than their private sector counterparts. The general health and well-being of private sector employees in Kerala may be greatly impacted by the low amount of old-age pension of ₹1600 (~19.11 USD) [9]. The low level of financial support and financial instability may raise stress and anxiety levels, which can worsen health conditions and lower both mental and physical well-being. In addition to limiting access to healthy food, vital healthcare services, and social interaction opportunities, financial limitations can also negatively impact an individual's quality of life and general health. Studies having similar results show that employees of the public sector can better prepare for the transition since they frequently have access to more organized retirement planning resources and explicit retirement age rules [10].

This finding suggests a need for policymakers to consider revising and increasing the pension amounts for private sector employees to ensure greater financial security post-retirement. Introducing a minimum pension guarantee scheme for private sector retirees would help alleviate financial stress and improve overall mental well-being. Other factors, such as improved pensions that are steadier and more predictable, better health benefits after retirement, and increased respect and social recognition that come with working for the public sector, can be credited for this. On the other hand, retired private sector employees frequently experience financial instability as a result of less stable pension plans and inadequate retirement savings, which can lead to higher levels of stress, worry, and depression [11]. Due to formal retiree clubs and government-sponsored community events, public sector retirees frequently continue to participate in more social interaction. Retirement from the private sector may not have the same structured social networks, which could result in social exclusion and loneliness [12]. There is a need for further research to ascertain how the adoption of a transition plan might affect retirement decisions and related mental health outcomes, as the existing literature is inconclusive about the effects of retirement on mental well-being [13].

Participants aged above 68 years were found to have a 1.36 times higher likelihood of experiencing lower mental well-being compared to those aged 68 years and younger. This decline in mental well-being among older adults aligns with broader trends observed in the literature, where aging is associated with increased risks of mental health issues such as depression and anxiety. The WHO notes that around 14% of adults aged 60 years and over live with a mental disorder, and this age group is particularly vulnerable to factors like loneliness, social isolation, and the cumulative impacts of earlier life experiences, which can exacerbate psychological distress​ [14,15].

Further, the Centers for Disease Control and Prevention (CDC) highlights that old-olds often face significant life changes, such as retirement, loss of loved ones, and declining physical health, which contribute to decreased mental well-being. These stressors can lead to increased symptoms of depression and anxiety, as well as other mental health conditions​ [16]. Also, social support plays a crucial role in maintaining mental well-being among older adults, and the lack of such support for old-olds can further deteriorate their mental health​ [17].

In this study, rural participants' mental well-being was lower than that of urban participants. This might be due to social connections and support from the urban area and economic stability among urban participants. A study from China reported a lack of access to mental health resources as one of the main causes of rural retirees’ decreased mental well-being [18]. Rural communities may have fewer mental health specialists, facilities, and services than urban regions, which are likely to increase untreated mental health concerns [18,19]​. Furthermore, several social network characteristics vary depending on the region. In particular, individuals from rural areas showed greater diversity in the ages within their social networks, both in terms of the age similarity between them and their social ties and in terms of the age similarity across their social links. This may be because there are fewer individuals living in rural regions, which narrows the social pool and encourages adults living in rural areas to seek out social connections with people [20].

This study found that retired public sector employees had a higher quality of life than retired private sector employees. Some studies have similar reports describing that secure and larger pensions are provided to public sector retirees, improving their financial stability and reducing their stress levels. Additionally, better healthcare access is ensured for retired public sector employees, who typically have access to comprehensive health insurance coverage. However, private retirees sometimes have little to no access to these benefits, which increases their out-of-pocket expenditure and limits their ability to receive essential medical care [21]. A retirement plan for public sector employees ensures that individuals age with dignity and maintain their current level of living. Pension plans offer the chance to save and invest, and when one retires, one can use an annuity plan to get a lump sum payment as regular income [22].

In this study, it was found that the participants aged 69 years and older had a poorer QoL compared to those aged 68 years and younger. This observation aligns with previous research studies, which indicated a decline in QoL with advancing age in this population [23,24]. This diminished QoL among older retirees may be attributed to their increased vulnerability to the challenges of sudden life changes and a reduction in social activity. Continued employment can provide stability in lifestyle, a consistent income, and opportunities for social interaction, all of which contribute to a better QoL. Therefore, preserving employment status or engaging in meaningful activities post-retirement is essential for supporting the QoL of older adults, helping them stay socially connected and financially secure [25].

The current study highlights a gender difference in QoL among retired employees, with males having better QoL, similar to patterns observed in other low- and middle-income countries (LMICs). Research from various LMICs consistently reports that male retirees generally experience a better QoL compared to their female counterparts. This disparity is attributed to distinct cultural norms and social influences that men and women are subjected to. Earlier studies indicate that female participants often have a lower social standing, face greater financial constraints, and encounter more obstacles in accessing healthcare services. Additionally, women tend to have increased domestic duties, which further limits their opportunities for leisure and self-care. These factors collectively contribute to the observed gender disparity in QoL, underscoring the need for targeted interventions to address these inequalities [26].

In this study, it was observed that participants from rural regions and those belonging to households below the poverty line (BPL) had a poorer QoL. Rural areas, often reliant on resource extraction and agriculture, tend to offer less diversity and stability in income [27], which may contribute to the lower QoL among these residents. However, a few studies [28,29] have reported a better QoL for individuals residing in rural areas. The discrepancies in these findings may be attributed to differences in the populations studied and variations in study design or methods of analysis. Similarly, BPL participants experience greater financial stress compared to their APL counterparts, who typically have the means to afford better housing, food, and other essentials. This financial disparity significantly impacts the living conditions and overall well-being of these groups. BPL individuals often struggle with limited resources, making it challenging to secure adequate housing, access nutritious food, and afford healthcare. In contrast, APL individuals generally enjoy more stable financial conditions, which allow them to live in safer, better-maintained housing and have access to higher-quality necessities [30]. The financial instability faced by BPL households is closely linked to diminished health and general well-being. The inability to meet basic needs can lead to poor physical health outcomes, as well as increased mental stress and anxiety. These challenges underscore the critical role that economic conditions play in shaping QoL. Financial stability, as seen in APL households, provides a buffer against many of these stressors, contributing to better health and a higher QoL [31].

Study limitations

To the best of our knowledge, this is one of the first studies to compare the mental well-being and QoL of retired public and private employees. However, there are a few limitations. The study was constrained by its cross-sectional design, which limits its ability to establish causal associations between the identified factors and the mental well-being or QoL of the participants. This study relied on self-reported data, which could introduce response bias, as participants might have provided socially desirable answers or may not have accurately remembered past details, potentially affecting the validity of the findings. Additionally, as the research was conducted in only one district of Kerala and within a limited time period, the results may not be generalizable to other regions or the entire population of retired employees.

## Conclusions

Retired public sector employees have higher mental well-being and higher QoL compared to retired private sector employees. This highlights the need for enhanced retirement policies and support systems that are specifically tailored to address the unique challenges faced by retired individuals from the private sector. Enacting comprehensive strategies and laws that prioritize mental well-being, ensure financial stability, and promote active engagement in society can significantly improve the quality of life of retired employees from the private sector. Policymakers should consider increasing pension amounts under schemes like the Indira Gandhi National Old Age Pension Scheme, expanding the Vayomithram initiative to rural areas, and enhancing healthcare access through subsidized health insurance programs tailored to private sector retirees in Kerala. Furthermore, offering readily available mental health services, assistance with financial planning, and avenues for fostering community connections could aid in reducing the disparity in mental well-being between these populations.
